# Supplementary material for: MONET: Multi-omic module discovery by omic selection
Source: PLoS Comput Biol. 2020 Sep 15;16(9):e1008182. doi: 10.1371/journal.pcbi.1008182 (PMC7518594; doi:10.1371/journal.pcbi.1008182)
Supplement: S1 Appendix — (DOCX) [file pcbi.1008182.s001.docx]

Appendix for“MONET: Multi-omic patient module detection by omic selection”

Nimrod Rappoport, Roy Safra and Ron Shamir^*^

The Blavatnik School of Computer Science, Tel Aviv University, Tel Aviv 69978, Israel

*To whom correspondence should be addressed. Tel: +972 3 640 5383; Fax: +972 3 640 5384;

Email: [rshamir@tau.ac.il](mailto:rshamir@tau.ac.il) (RS)

Contents

[MONET's second weighting scheme and probabilistic formulation 2](#_Toc38486285)

[Simulations 4](#_Toc38486286)

[Simulation I 4](#_Toc38486287)

[Simulation II 4](#_Toc38486288)

[Datasets 5](#_Toc38486289)

[Image dataset 5](#_Toc38486290)

[scNMT 5](#_Toc38486291)

[Breast Invasive Carcinoma microarray 5](#_Toc38486292)

[Benchmarked methods and software 6](#_Toc38486293)

[Hardware 8](#_Toc38486294)

[Supplementary Tables 9](#_Toc38486295)

[Supplementary Figures 13](#_Toc38486297)

[Bibliography 20](#_Toc38486298)

# MONET's second weighting scheme and probabilistic formulation

As discussed in the main text, MONET can use different weighting schemes to calculate the edge weights in the omic graphs. We now present another weighting scheme, which provides a probabilistic interpretation to MONET. This weighting scheme is based on a similar formulation from MATISSE [1], which in turn builds on a similar idea from CLICK [2].

Recall that we compute the similarity (e.g. correlation) between every pair of samples $u, v$ in every omic $l$, denoted $sim_{l}(u, v)$. In this weighting scheme, we assume that every two samples are either similar to one another, in which case these samples are called "mates", or are not similar, in which case they are "non-mates". In general, mate samples should belong to the same module in omic $l$, and non-mate samples should belong to different modules. We assume that similarity values between mates originate from one normal distribution, and similarity values between non-mates originate from another normal distribution (with a possibly different variance). This modeling has theoretical justifications in certain conditions [2]. The observed set of similarity values, $\left\{ sim_{l}\left( u, v \right) \right|u and v are samples\}$, is therefore a mixture of two Gaussians.

Assume we already know the parameters of the Gaussian mixture model. For two samples $u$ and $v$ we can now assign weights to the edges in the omic graph as follows:

$$w_{l}\left( u, v \right)=log(\frac{\Pr\left( sim_{l}(u, v) | u and v are mates \right)}{\Pr\left( sim_{l}(u, v) | u and v are non-mates \right)})$$

Using this formulation, the weight of a module is now:

$$weight\left( M \right)=\Sigma_{l\in omics\left( M \right)}\Sigma_{u, v\in samples\left( M \right)}w_{l}\left( u, v \right)= \Sigma_{l\in omics\left( M \right)}\Sigma_{u, v\in samples\left( M \right)}log(\frac{\Pr\left( sim_{l}(u, v) | u and v are mates \right)}{\Pr\left( sim_{l}(u, v) | u and v are non-mates \right)})$$

The weight of the module $M$ is therefore the score for a log-likelihood ratio test for whether $samples\left( M \right)$ form a module on $omics\left( M \right)$, under the simplifying assumption that modules and sample pairs are independent. A positive weight indicates that this set of samples is likely to form a module on the set of omics. Modules with high positive weight therefore correspond to likely modules under a hypothesis-testing framework.

To learn the parameters of the model, we used a mixture of Gaussians EM algorithm (implemented in the mixtools R package). We performed 20 repeats, chose the most likely parameters across all the runs, and calculated $w_{l}\left( u, v \right)$ using the obtained probabilities. For every omic, we used the eigengap method on the omic similarity matrix to estimate the number of clusters. If the estimated number of clusters for omic $l$ is $num\_clusters_{l}$, denote by $C$ the $1-1/num\_clusters_{l}$ quantile of the similarity values. We performed the update $w_{l}\left( u, v \right)= w_{l}\left( u, v \right)-C$. The rationale behind this update is that if the estimated number of clusters is 5, after the update 1/5 of the edges in the graph will be positive, which is an estimation for the number of edges in the graph between mates. Note that adding constants to the edge weights has the probabilistic interpretation of adding a Bayesian prior to the probability that two samples are mates.

More generally, for a pair samples $u, v$ we define a binary variable $A_{l}(u, v)$ to indicate whether the samples belong to the same module in omic $l$ or not. If $sim_{l}(u, v)$ follows different distributions in the case $A_{l}(u, v)$ is true and in the case it is false, we can set:

$$w_{l}\left( u, v \right)=log(\frac{\Pr\left( sim_{l}(u, v) | A_{l}(u,v) \right)}{\Pr\left( sim_{l}(u, v) | \bar{A_{l}\left( u,v \right)} \right)})$$

And the weight of a module $M$ will be as before the score for a log-likelihood test for whether the module's samples really form a module.

We executed this weighting scheme on all TCGA datasets, and found a significant association with survival in 6 solution, and a clinically enriched parameters in 5. We therefore recommend using the weighting scheme presented in the main text, except for classification, where the weighting scheme that is based on consensus clustering cannot be used.

# Simulations

## Simulation I

We simulated samples from five modules, each with 60 samples, and two omics of dimension 500. Module 1 covers only omic 1, module 2 only omic 2, and modules 3-5 cover both omics. Each module has a *center* in each omic that it covers, and each sample is drawn from a normal multi-variate distribution around that center with unit covariance matrix. The center of each module was a vector of length 500 of all zeroes, except for 125 entries that equal 1 and characterize this module. The 125 module-specific features were disjoint for each module. The center of module 1 in omic 2 likewise used 125 entries that equal 1, but these entries were randomly sampled, and the covariance matrix of the normal distribution equaled $4*I$, where $I$ is the identity matrix. Module 2 in omic 1 was generated in a similar manner. Five outlier samples were generated using multi-variate distribution with mean $\vec{0}$ and covariance $0.1*I$.

## Simulation II

We simulated samples from five modules, each with 30 samples, and three omics of dimension 500. As in simulation I, each module was simulated using multi-variate normal distribution in each omic that it covers. Module 1 had 100 non-zero entries in its center in omic 1. Modules 2-4 all had the same center in omic 1, with 100 non-zero entries. In omics 2 and 3, all modules had different centers, with different non-zero entries. In omic 2 each module had 20 non-zero entries, creating a weak clustering structure, and in omic 3 each had 40 non-zero entries.

# Datasets

## Image dataset

The dataset was downloaded from: <https://archive.ics.uci.edu/ml/machine-learning-databases/mfeat/> On 23 November 2018. It contains 2000 images of the digits 0-9, each digit with 200 images. Each image has the following 6 omics:

1. mfeat-fou: 76 Fourier coefficients of the character shapes;
2. mfeat-fac: 216 profile correlations;
3. mfeat-kar: 64 Karhunen-Love coefficients;
4. mfeat-pix: 240 pixel averages in 2 x 3 windows;
5. mfeat-zer: 47 Zernike moments;
6. mfeat-mor: 6 morphological features.

## scNMT

Data was downloaded from:
<https://github.com/BIRSBiointegration/Hackathon/tree/master/scNMT-seq>

## Breast Invasive Carcinoma microarray

Agilent mRNA expression microarray data was downloaded from:
<http://firebrowse.org/?cohort=BRCA&download_dialog=true>

# Benchmarked methods and software

Experiments were run on R version 3.5.2 64 bit.

For all methods, sequencing data (either RNA-seq or miRNA-seq) was log-transformed and features with 0 variance were removed. SNF, MONET and NEMO used all features. For MDI, clusternomics and MOFA+ in each omic only the 2000 most highly variable features were kept. For BCC and TWL only 500 features were kept due to runtime. Each feature was then normalized to have mean 0 and standard deviation 1.

MONET – MONET was executed as described in the main text. In the image dataset all methods clustered the data into 10 clusters. MONET cannot get as input the number of clusters. Instead, when using NEMO to compute the edge weights, NEMO clustered each subsampled omic into 10 clusters, and the edge weights of each graph were shifted such that 10% of the weights were positive. This approach still does not guarantee that MONET finds the desired number of clusters.

To apply MONET to the scNMT data, we counted for every cell the number of promoters for which methylation was not measured. We removed the 25% of cells for which this number was lowest, and for every other cell we set to NA the methylation status for randomly selected promoters, such that all cells had the same number of measured promoters. We executed MONET as described before.

To use MONET to discover gene modules, we only kept genes that were among the top 2000 most highly variable features in both the RNA-seq and microarray data. This left 1532 genes. Each gene was then normalized to have mean zero and standard deviation 1 in each omic. These omic matrices were then transposed and given as input to NEMO (which then normalized the *samples* to mean 0 and standard deviation 1, as it always does to its input).

As mentioned in the main text, MONET's algorithm for finding heavy subgraphs was run 15 times in parallel unless otherwise specified, and the solution with the highest score was returned. The runs were made on up to 20 cores simultaneously.

SNF – SNF was executed as described previously in Rappoport et al., 2018 [3].

NEMO – NEMO was executed as described previously in Rappoport et al., 2019 [4]. Default parameters were used. When NEMO was run on single-cell data with many NA values per cell, as part of MONET's edge weight calculation, only values that are not NA were used to calculate distances between cells.

BCC – We used the R package bayesCC available here: <https://github.com/ttriche/bayesCC>. We executed BCC in parallel for K clusters with K ranging from 2 to 15. For each K, we executed the function bayesCC with IndivAlpha=TRUE and a maximum of 10,000 iterations. We selected the solution with highest mean adherence. Execution time reported is the wall clock time.

MDI –We used a recent implementation of MDI (mdipp-1.0.1) available here: <https://warwick.ac.uk/fac/cross_fac/zeeman_institute/zeeman_research/software/>. We executed the binary without CUDA support. To parse the output of the binary, determine the number of clusters, and perform the clustering, we used R scripts available here: <https://github.com/cyversewarwick/mdipp/>. Execution time reported only considers the data normalization, the execution time reported by the MDI binary, and the output parsing. The time taken to write to disk the input to the binary is not included.

Clusternomics – We used the R package clusternomics available here: <https://github.com/evelinag/clusternomics>. We executed clusternomics with either 3 or 5 clusters per omic, and with 5, 10, …, 30 global clusters, as performed in the authors' analysis. All these executions were done in parallel. For each of these options we called the function contextCluster with a maximum of 10,000 iterations, 3 iteration lag, 5000 iterations burn-in, and while modeling the data as normal with diagonal covariance matrix. We chose the clustering solution with minimal deviance information criterion. Execution time reported is the wall clock time.

MOFA+ – We used the R package available here: <https://github.com/bioFAM/MOFA2>. We executed MOFA+ using the function prepare_mofa with the default data options, model options and train options. We used 42 as a random seed and allowed up to 10000 iterations. The number of factors we used is the minimum between 15 and the minimal number of features in any of the omics. We then used k-means on the factors (with 30 random starts and up to 10000 iterations) to cluster the samples into 2,…,15 clusters, and chose the clustering solution with maximal silhouette score.

TWL – We used the R package available here: <https://cran.r-project.org/web/packages/twl/index.html>. We used a maximum of 10,000 iterations, where 2,000 were used as burn-in. Since the number of samples and omics we used was similar to the numbers used in TWL's original analysis, we used the same values for $\alpha$ and $\beta$, except for the digit dataset were we used $\beta=0.4*2$ because it has twice the number of omics. TWL only returns a clustering per omic, so we tried to different approaches to create global clusters. In the first we looked at the Cartesian product of omic-specific clusters. This approach resulted in a very high number of clusters and poor results (data not shown). TWL outputs per omic a matrix which signifies the posterior probability that each pair of samples belong to the same cluster in that omic. We averaged these matrices across all omics, and continued with TWL's clustering process, but assuming a single-omic whose posterior probabilities is given by the matrix we calculated. We determined the number of clusters in the data using the eigengap method [5] on the matrix of average posterior probabilities.

# Hardware

All experiments for timing the different methods were performed on a cluster:

Linux 4.9 72 CPUs, 2300 MHz each 756 GB RAM 64 bit operating system.
Since several methods use parallelization, the presented time for all methods is the wall clock time.

# Supplementary Tables

Table A - TCGA log-rank p-values

##

Table B - TCGA number of enriched clinical parameters

Table C - TCGA runtime (seconds)

Table D - TCGA number of clusters

Table E – venous invasion status for modules in MONET's solution on Ovarian Serous Cystadenocarcinoma data from TCGA

Table F - mutations tested for enrichment in MONET's solution on Ovarian Serous Cystadenocarcinoma data from TCGA

Table G – highly expressed genes in Module 3 of MONET's solution on Ovarian cancer.

Table H – Mouse embryonic day of development for MONET's solution on scNMT data.

Table I – Cell types for MONET's solution on scNMT data.

Table J – Mouse embryonic day of development and cell type for MONET's solution on scNMT data.

# Supplementary Figures


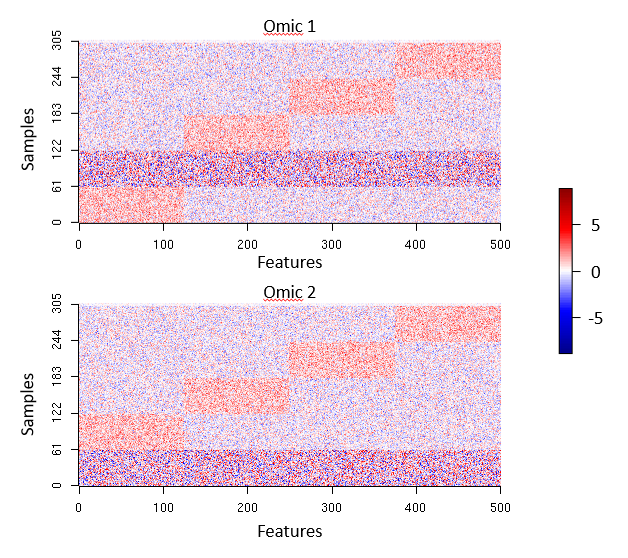


Figure A – raw data for simulation I. Rows are samples and columns are features. Samples 1-60 are M1, 61-120: M2, …,241-300: M5, 301-305: lonely.


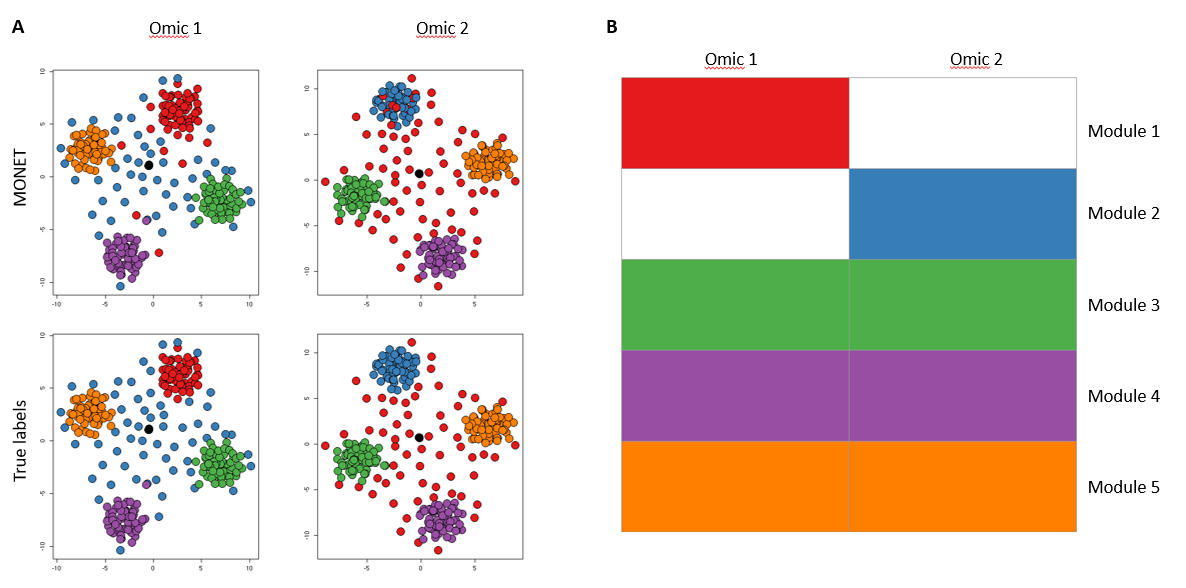


Figure B – MONET's results in simulation I. A: t-sne visualization of the raw data in simulation I. Samples are colored by MONET's output (top) or the true labeling (bottom). Lonely samples are colored in black. B: Module omics identified by MONET on simulation I. Each row signifies a module and each column an omic. Colored panels indicate that the omic is covered by the module, white indicates that it is not.


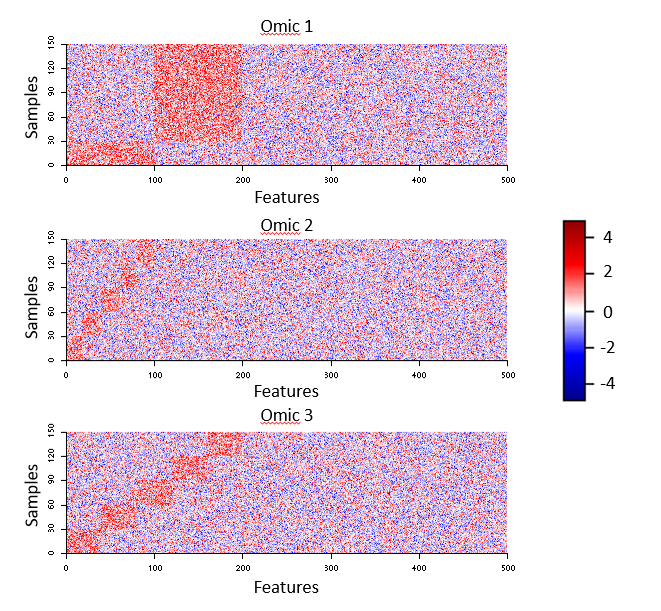


Figure C – raw data for simulation II. Rows are samples and columns are features. Samples 1-30 are M1, 31-60: M2, …,121-150: M5.


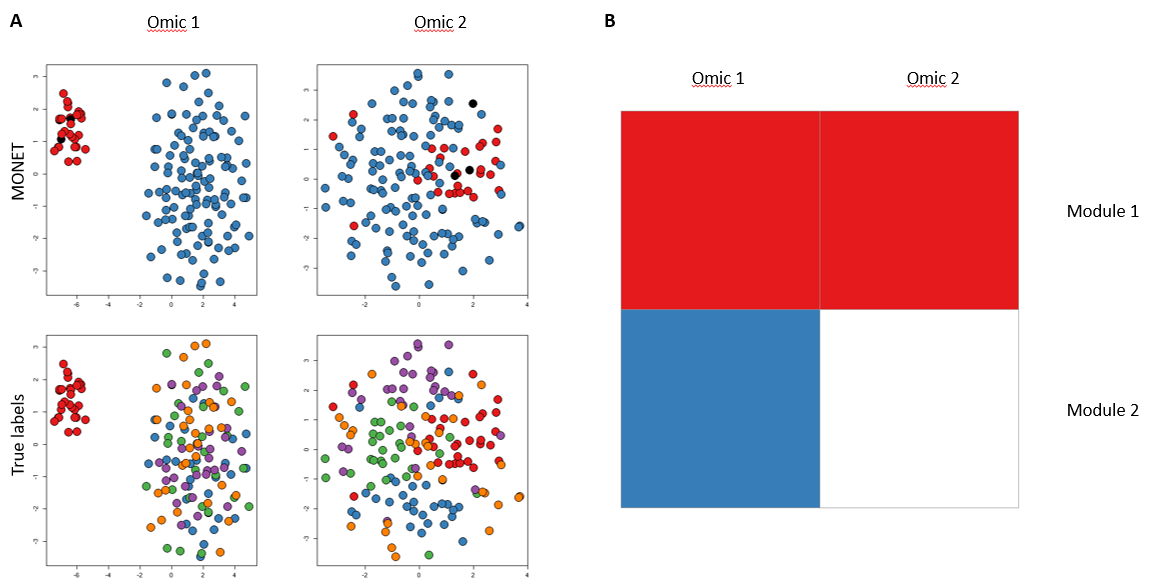


Figure D – MONET's results in simulation II when using only two first two omics. A: t-sne visualization of the raw data in simulation II using only the first 2 omics. Samples are colored by MONET's output (top) or the true labeling (bottom). B: Omics covered by each MONET module in simulation II when using only the first two omics. Modules are colored as in the top panels in A.


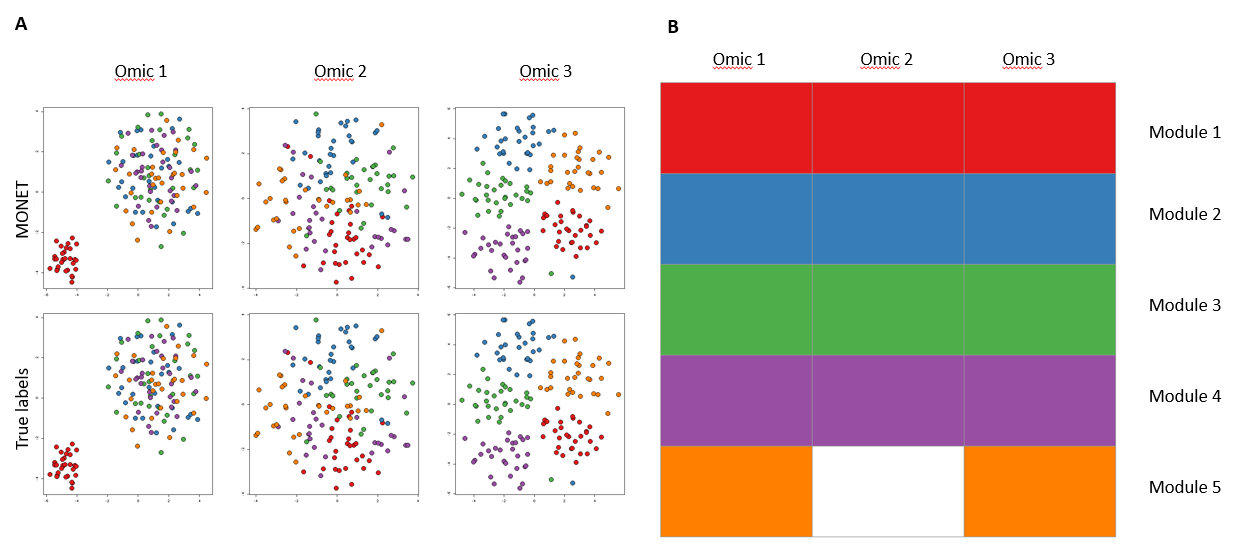


Figure E – MONET's results in simulation II when using all three omics. A: t-sne of raw data in simulation II using all 3 omics. Samples are colored by MONET's output (top) or the true labeling (bottom). B: Omics covered by each MONET module in simulation II when using all the three omics. Modules are colored as in the top panels in A.


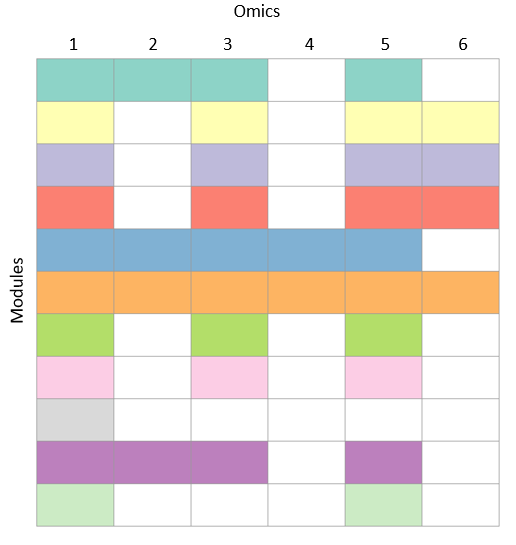


Figure F – Omics covered by each MONET module in the image dataset. Columns are omics and rows are modules.


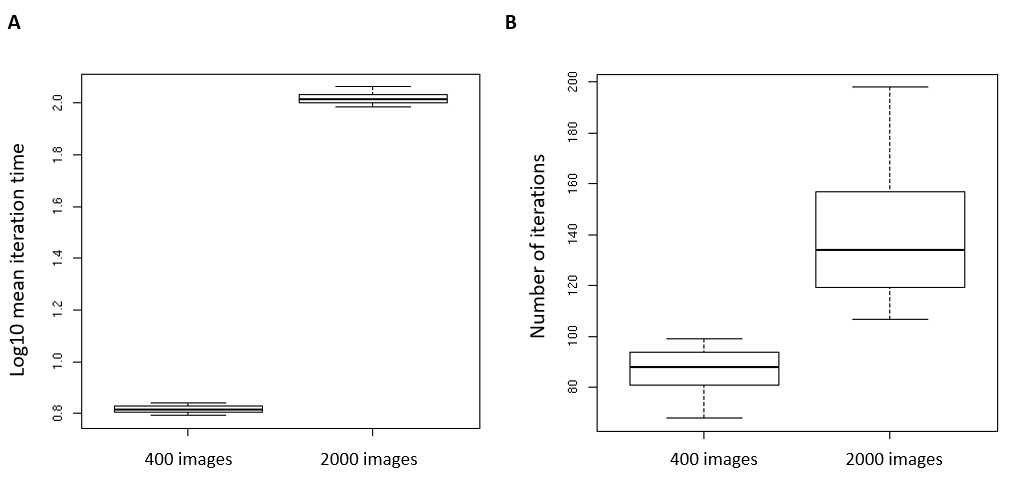


Figure G – Executing MONET on 400 and 2000 images. In both cases, MONET was executed 15 times, and for every execution the mean iteration time and the number of iterations were recorded. A: log10 mean iteration time (in seconds) across 15 executions. B: Number of iterations across 15 executions.


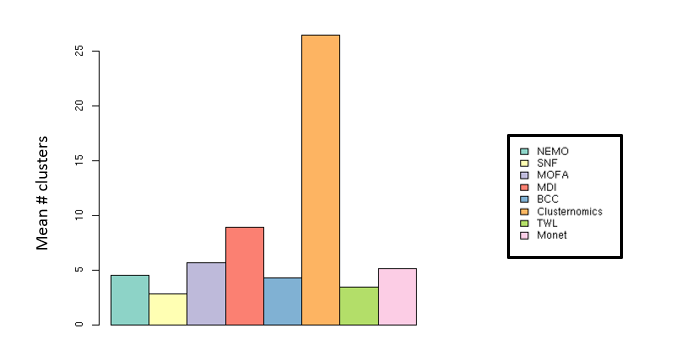


Figure H – Mean number of clusters chosen by each method across ten TCGA cancer datasets.


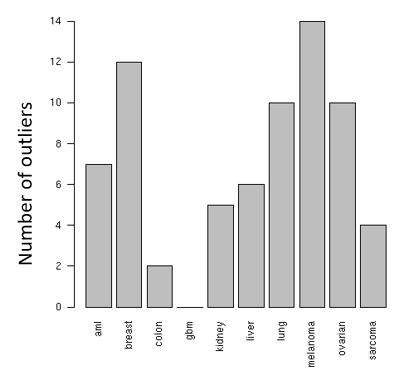


Figure I – Number of outliers (lonely samples) reported by MONET per cancer type.


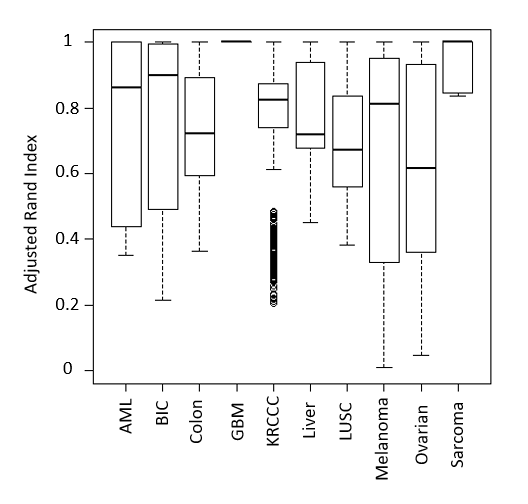


Figure J – Stability of MONET's solutions across ten TCGA datasets. For every dataset, the algorithm was run 50 times, and the distribution of Adjusted Rand Index across all pairs of solutions is shown.


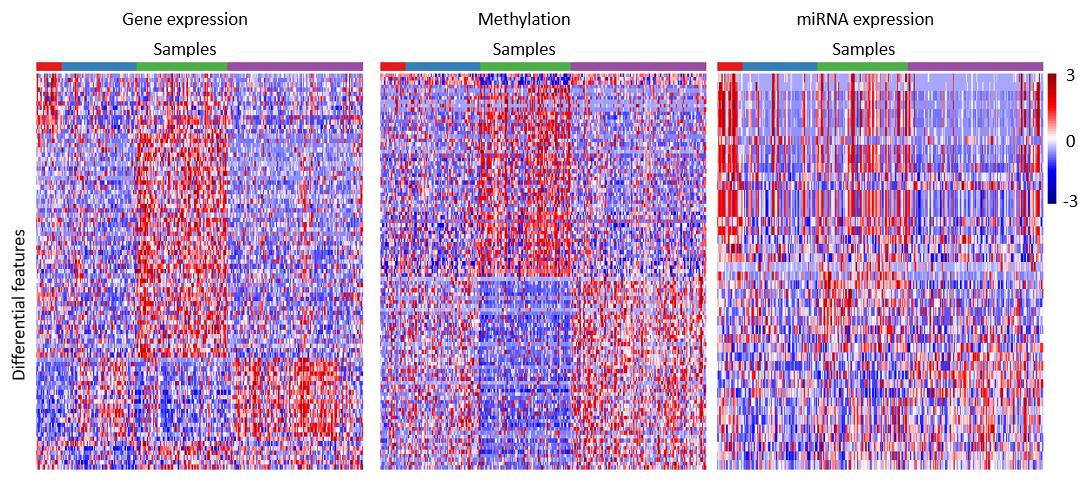


Figure K – Heatmaps showing differential features across three omics in the TCGA ovarian cancer dataset. Each panel represents one omic. The color bars indicate the modules to which each sample belongs (M1-M4 from left to right; 10 lonely samples not included). Values are normalized to have mean 0 and standard deviation 1 across samples.


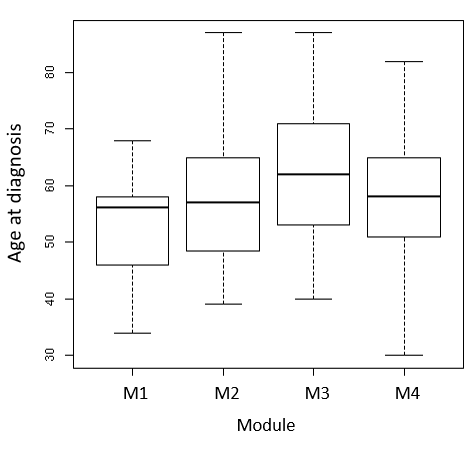


Figure L – Age distribution across 4 MONET modules for Ovarian cancer.


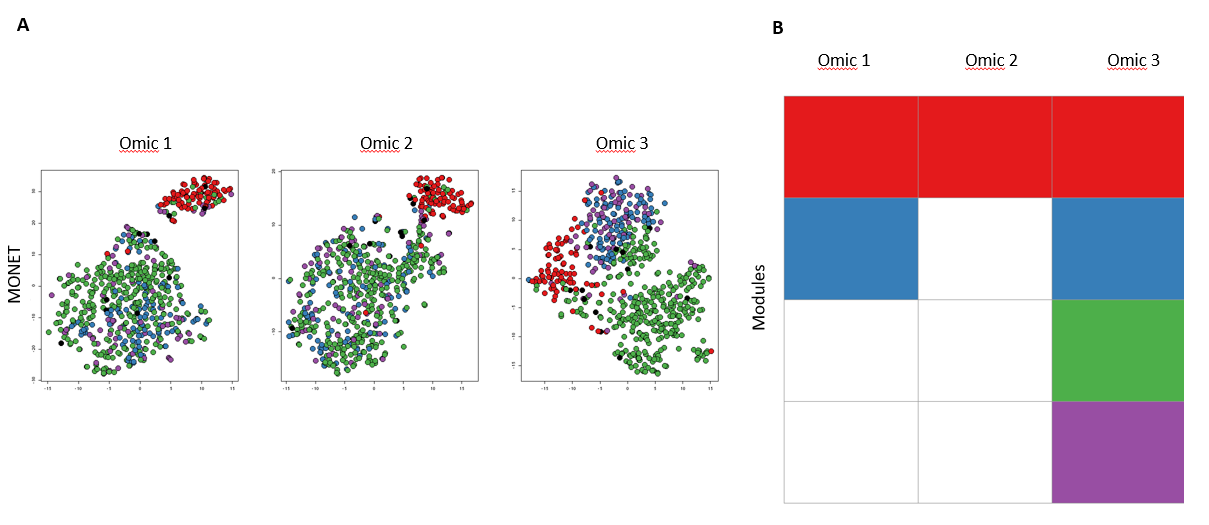


Figure M – MONET's results on breast cancer from TCGA. A: t-sne visualization of MONET's solution. Samples are colored by their MONET module. B: Omics covered by each MONET module. Columns are omics and rows are modules.


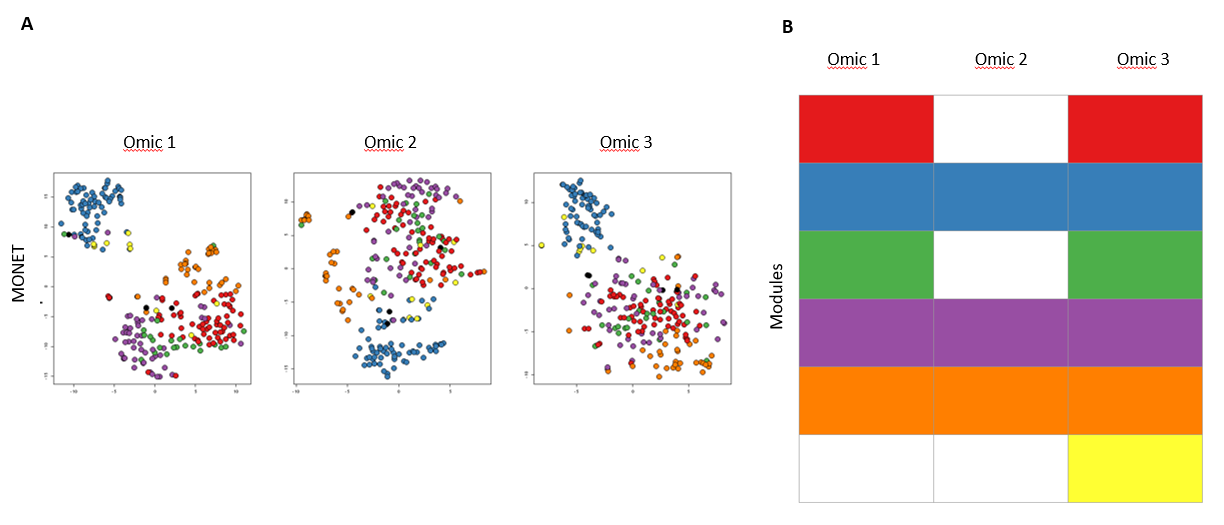


Figure N – MONET's results on Sarcoma from TCGA. A: t-sne visualization of MONET's solution. Samples are colored by their MONET module. B: Omics covered by each MONET module. Columns are omics and rows are modules.


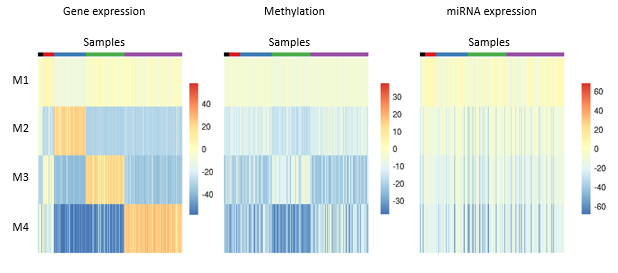


Figure O – Module association scores for MONET's solution of TCGA ovarian cancer dataset. Every panel shows the association between all samples (columns) and modules (rows) for a specific omic. The color bars indicate the modules to which each sample belongs (lonely, M1-M4 from left to right).


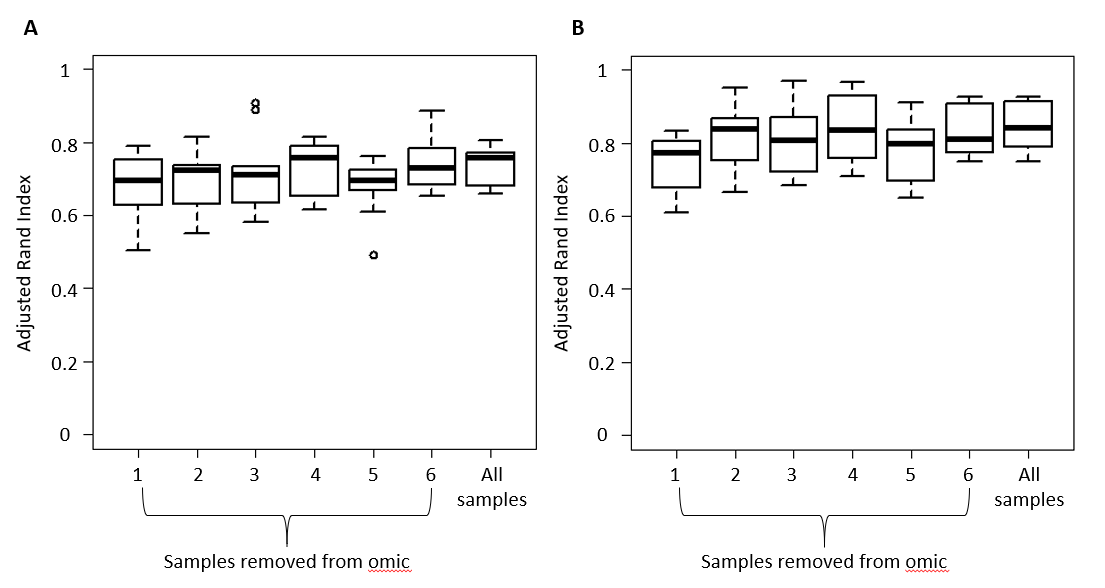


Figure P – ARI in partial datasets experiments for the image dataset. A: ARI distribution for samples dropped in each omic, and for all the samples in the dataset (rightmost box), compared to the ground truth solution. B: same as A, only comparing to the solution on all samples.


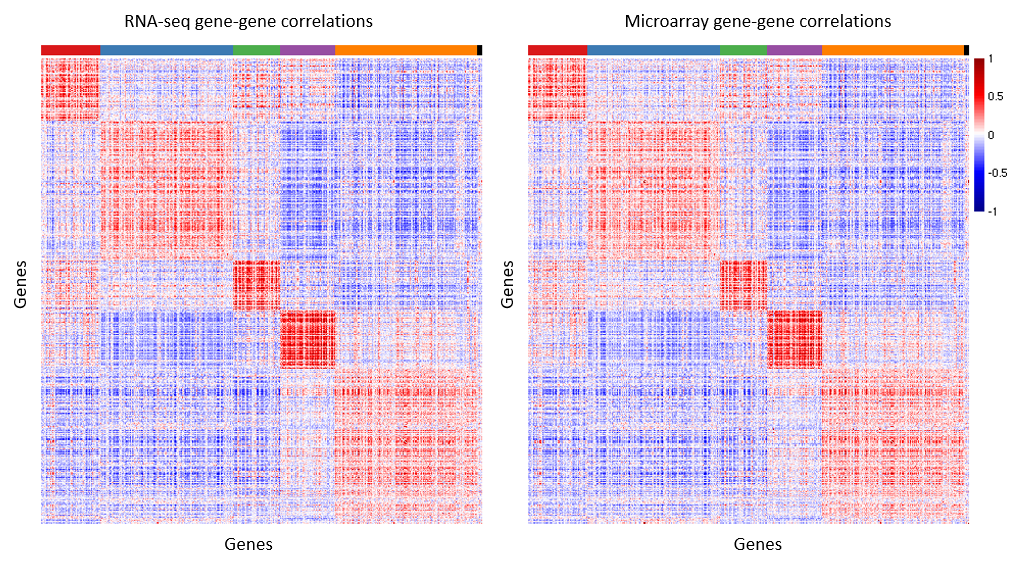


Figure Q – Correlation matrix of genes in the Breast Invasive Carcinoma dataset using RNA-seq (left) and microarrays (right). Genes are marked by their MONET module.

# References

1. Ulitsky I, Shamir R. Identification of functional modules using network topology and high-throughput data. BMC Syst Biol. 2007;1: 8. doi:10.1186/1752-0509-1-8

2. Sharan R, Shamir R. CLICK: A clustering algorithm with applications to gene expression analysis. AAAI Press; 2000. pp. 307–316.

3. Rappoport N, Shamir R. Multi-omic and multi-view clustering algorithms: review and cancer benchmark. Nucleic Acids Res. 2018;46: 10546–10562. doi:10.1093/nar/gky889

4. Rappoport N, Shamir R. NEMO: cancer subtyping by integration of partial multi-omic data. Schwartz R, editor. Bioinformatics. 2019;35: 3348–3356. doi:10.1093/bioinformatics/btz058

5. von Luxburg U. A tutorial on spectral clustering. Stat Comput. 2007;17: 395–416. doi:10.1007/s11222-007-9033-z
