# Supplementary material for: MONET: Multi-omic module discovery by omic selection
Source: PLoS Comput Biol. 2020 Sep 15;16(9):e1008182. doi: 10.1371/journal.pcbi.1008182 (PMC7518594; doi:10.1371/journal.pcbi.1008182)
Supplement: Attachment — Submitted filename: itemized_response_to_reviews.pdf [file pcbi.1008182.s003.pdf]

Itemized response to PLOS Computational Biology reviews of:

# "MONET: Multi-omic module discovery by omic selection", by N. Rappoport, R. Safra and R. Shamir

We thank the authors for their positive and valuable reviews. We addressed the points raised by the reviewers. In addition to these points, we also changed the coloring of the right panel of Figure 5B, where we previously used an incorrect color code.

Reviewer #1: The clarity of the exposition has been substantially improved, and the authors have responded to all of my points adequately.

We thank the reviewer for the evaluation.

There are only a couple of outstanding issues:

- Unlike other methods, MOFA+ is not mentioned in the Introduction.

Response: We now mention MOFA+ in the introduction.

- I still believe there is an overstatement of the meaning of the results (pg. 8): "different omics do have different structures". This refers to MONET's solutions on the cancer data, but MONET is designed to capture such signal. In the absence of a ground truth, I do not believe that such a strong statement can be made: it is possible that MONET's increased clustering flexibility is leading it to overfit and identify spurious correlations resulting from the choice of similarity function and optimisation objective.

These results suggest that different omics may have different structures.

Response: We changed the phrasing to the one suggested by the reviewer, and changed an additional similar statement on pg. 7.

- Regarding the significance of the survival curves of Figure 3C, it seems that M2 is the sole driver of that statistical significance. Without it, the MONET's modules would not show differential survival. Again, it seems that the text is overly optimistic.

Response: The text currently presents the survival analysis for Figure 3C as follows: "The modules showed significant differential survival (p=0.036, Fig 3C), with M2 showing significantly better survival than the others (p=4e-3)". We agree with the reviewer that the significant difference in survival is due to M2's favorable survival, but we think the text already conveys this notion.

Reviewer #2: I appreciate the authors' effort to revise and improve this manuscript. The presentation has been strengthened. The authors also added more analysis. Overall, this is a good method contribution with meaningful applications to cancer genomic data and potentially other multi-omics datasets.

We thank the reviewer for the positive evaluation.

I only have one remaining question that I hope the authors can clarify with either more comparison or at least more method comparison discussions.

It appears that the problem can also be approached by using non-negative tensor factorization. As a special case, this will be non-negative matrix factorization where there are quite a few earlier work in trying to identify the latent variables and also the hidden structures for the clusters, e.g., Zhang et al. (PMID: 22879375) Liu et al. (PMID: 24491042) and Shen et al. (PMID: 19759197). I suggest that the authors put MONET in the context of these related prior work and clarify the advantage.

Response: Shen et al. (or more accurately, iClusterBayes, which is iCluster's latest version) was included in our previously published benchmark of multi-omic clustering algorithms. The benchmark, which is cited in our paper, used the same data as here. It also included an additional NMF-based method (MultiNMF, by Liu et al. 2013). MONET outperformed all methods that were included in the benchmark, including iClusterBayes and MultiNMF. We now mention these methods in the introduction and on page 7 emphasize that they were included in our previous benchmark.

Zhang et al. is used primarily for clustering features from different omics (e.g. genes in gene expression and CpG sites from methylation data). Liu et al. does detect cancer subtypes, but using gene expression and prior network data. We focused specifically on methods for multi-omic patient clustering, and therefore did not include these works.
